# Exploratory Study of Clinical Factors Associated with MPO-ANCA Positivity in Nontuberculous Mycobacterial Pulmonary Disease

**DOI:** 10.3390/jcm15155794

**Published:** 2026-07-24

**Authors:** Hidenori Takahashi, Yugo Satake, Takumi Yasuda, Kota Taguchi, Kanako Furukawa, Hiroki Nagamatsu, Ryutaro Hirose, Naoya Toba, Mio Toyama-Kousaka, Shinichiro Ota, Miwa Morikawa, Masaharu Shinkai

**Affiliations:** Department of Respiratory Medicine, Tokyo Shinagawa Hospital, 6-3-22, Higashi-Oi, Shinagawa-ku, Tokyo 140-8522, Japanshin.ohta0915@gmail.com (S.O.);

**Keywords:** nontuberculous mycobacterial pulmonary disease, MPO-ANCA, ANCA-associated vasculitis, *Mycobacterium avium* complex, MAC antibody

## Abstract

**Background/Objectives:** Nontuberculous mycobacterial pulmonary disease (NTM-PD) has been associated with myeloperoxidase anti-neutrophil cytoplasmic antibody (MPO-ANCA)-positive ANCA-associated vasculitis (AAV). However, clinical factors associated with MPO-ANCA positivity in patients with NTM-PD remain unclear. This study determined the frequency of MPO-ANCA positivity among tested patients with NTM-PD and explored the clinical factors associated with MPO-ANCA positivity. Methods: This study retrospectively analyzed patients aged ≥15 years old with NTM-PD who were treated at Tokyo Shinagawa Hospital and underwent MPO-ANCA testing between April 2020 and March 2024. Clinical data, including nontuberculous mycobacterial species, serum *Mycobacterium avium* complex (MAC) antibody titers, radiographic findings, and nodule, infiltration/consolidation, cavity, and ectasis scores, were collected from their electronic medical records. Patients were classified into MPO-ANCA-positive and negative groups, and clinical factors were compared. Results: Among 109 patients with NTM-PD, 49 underwent testing for MPO-ANCA and proteinase 3 (PR3)-ANCA. MPO-ANCA was positive in five patients (10.2%), whereas PR3-ANCA was positive in one (2.0%). Among the five MPO-ANCA-positive patients, three had MAC antibody titers above the upper limit of measurement (>10 U/mL), and one developed AAV with biopsy-proven pauci-immune crescentic glomerulonephritis and mononeuritis multiplex. The MAC antibody titer was significantly higher in the MPO-ANCA-positive group than in the MPO-ANCA-negative group [10.0 (3.89–10.0) vs. 1.92 (0.19–6.19) U/mL, *p* = 0.038]. Conclusions: MPO-ANCA positivity was observed in a subset of tested patients with NTM-PD and was associated with higher MAC antibody titers. These findings suggest that chronic MAC-related antigen exposure or local pulmonary disease activity in NTM-PD may be associated with MPO-ANCA positivity.

## 1. Introduction

Anti-neutrophil cytoplasmic antibody (ANCA)-associated vasculitis (AAV) is an autoimmune disease characterized by necrotizing inflammation predominantly affecting small vessels and can cause multi-organ damage involving the kidneys, lungs, peripheral nerves, and other organs. Among ANCAs, myeloperoxidase (MPO)-ANCA is strongly associated with microscopic polyangiitis (MPA) [1].

Several case reports have described the development of MPO-ANCA-positive AAV after infection with nontuberculous mycobacteria (NTM). In our previous literature review, we reported that NTM-associated AAV can involve NTM species other than the *Mycobacterium avium* complex (MAC) and that some patients develop severe organ involvement, including rapidly progressive glomerulonephritis [2]. Recently, Oda et al. conducted a retrospective study of 63 patients with nontuberculous mycobacterial pulmonary disease (NTM-PD) and reported that 11.1% were ANCA-positive, most of whom were MPO-ANCA-positive, and 71.4% of the ANCA-positive patients developed AAV [3].

The mechanisms underlying ANCA induction in NTM infections remain unclear; however, chronic airway inflammation may provide persistent local immune stimulation that promotes neutrophil activation and neutrophil extracellular trap (NET) formation, thereby increasing exposure to neutrophil granule antigens, particularly MPO [1,4]. However, ANCA is not induced in all patients with NTM disease, and the clinical factors associated with MPO-ANCA positivity remain unclear. Therefore, this exploratory study aimed to determine the frequency of MPO-ANCA positivity among patients with NTM-PD who underwent MPO-ANCA testing at our hospital and to identify the clinical factors associated with MPO-ANCA positivity.

## 2. Materials and Methods

### 2.1. Study Design and Patients

This retrospective study included patients aged ≥15 years old with NTM-PD who were treated at Tokyo Shinagawa Hospital and underwent MPO-ANCA testing between April 2020 and March 2024. Follow-up data for MPO-ANCA-positive patients were reviewed through March 2024. NTM-PD was diagnosed according to the 2024 revised diagnostic criteria for NTM-PD proposed by the Japanese Society for Tuberculosis and Nontuberculous Mycobacteriosis and the Japanese Respiratory Society [5]. In accordance with the provisional diagnostic criteria for the initial diagnosis of pulmonary MAC disease, patients who met the clinical criteria and had a single positive sputum culture together with a positive serum anti-glycopeptidolipid-core immunoglobulin A (IgA) antibody test were also included [5,6,7].

### 2.2. Data Collection

For MPO-ANCA-positive patients, the index date was the first positive MPO-ANCA test date; for MPO-ANCA-negative patients, it was the MPO-ANCA measurement date used in the analysis. Clinical data at the index date, including age, sex, smoking status, body mass index (BMI), NTM species, history of standard multidrug treatment for NTM-PD, use of systemic immunosuppressive therapy, peripheral blood neutrophil count, serum albumin level, C-reactive protein (CRP), HbA1c, MAC antibody titer, MPO-ANCA, proteinase 3 (PR3)-ANCA, and radiographic findings, were retrospectively collected from electronic medical records. Smoking status was dichotomized as ever smoked or never smoked. History of standard multidrug treatment for NTM-PD was dichotomized as present or absent, with patients managed by observation alone or macrolide monotherapy alone classified as having no standard multidrug treatment history. Radiographic findings included the distribution of lung lesions, classified as unilateral or bilateral, and the nodule, infiltration/consolidation, cavity, and ectasis (NICE) score. MAC antibody titers exceeding the upper limit of measurement (>10 U/mL) were treated as 10 U/mL.

For patients with MPO-ANCA positivity, additional clinical details were reviewed descriptively, including pre-existing autoimmune diseases and available autoimmune serologic findings, the duration of NTM-PD, sputum acid-fast bacilli smear and mycobacterial culture results at the time of MPO-ANCA positivity, NTM treatment status, and longitudinal changes in MPO-ANCA levels and vasculitis-related clinical manifestations and outcomes.

### 2.3. Radiographic Assessment and NICE Score

Radiographic findings were evaluated using chest radiographs to assess disease distribution and the NICE score; chest computed tomography findings were also referenced when available. The NICE score was calculated according to the method reported by Kurashima et al. [8]. Each lung was divided into upper, middle, and lower zones, resulting in six zones. For each zone, the following four components were scored from 0 to 4: nodules, infiltration/consolidation, cavities, and ectasis. The total NICE score was calculated as the sum of the component scores, yielding a possible range of 0–96. Radiographic assessments and NICE score calculations were performed by a single respiratory physician.

### 2.4. Definition of MPO-ANCA and PR3-ANCA Positivity

In all analyzed patients, MPO-ANCA and PR3-ANCA levels were measured as a paired test at the same time point using a chemiluminescent enzyme immunoassay (CLEIA) at an external laboratory, SRL, Inc. (Tokyo, Japan). MPO-ANCA positivity was defined as a value > 3.4 U/mL, and PR3-ANCA positivity was defined as a value > 2.0 U/mL.

### 2.5. Statistical Analysis

First, patients with MPO-ANCA positivity were identified and their clinical characteristics, NTM species, radiographic findings, MAC antibody titers, sputum acid-fast bacilli results at the time of MPO-ANCA positivity, NTM treatment status, and AAV development were summarized. Subsequently, all analyzed patients were classified into MPO-ANCA-positive and MPO-ANCA-negative groups, and the clinical factors were compared between the two groups. Continuous variables were presented as medians with interquartile ranges and were compared using the Mann–Whitney U test. Categorical variables were presented as n/N (%) where appropriate and were compared using Fisher’s exact test. Statistical significance was defined as a two-sided *p*-value < 0.05. Statistical analyses were performed using EZR v.1.70 (Saitama Medical Center, Jichi Medical University, Saitama, Japan).

## 3. Results

### 3.1. Patient Characteristics and Frequency of MPO-ANCA Positivity

During the study period, 109 patients with NTM-PD visited our hospital. Among them, MPO-ANCA and PR3-ANCA levels were measured in 49 patients, who were included in the analysis. Among the tested patients, MPO-ANCA was positive in five patients (10.2%), whereas PR3-ANCA was positive in one patient (2.0%).

The clinical characteristics of the five MPO-ANCA-positive patients are shown in Table 1. The median age of the patients was 80 years, and all patients were female. None had a known autoimmune disease or was receiving systemic immunosuppressive therapy at the time of MPO-ANCA positivity. The causative NTM species were M. avium in three patients, mixed infection with M. avium and M. intracellulare in one patient, and M. kansasii in one patient. The radiographic patterns were bilateral nodular/bronchiectatic disease in four patients and bilateral fibrocavitary disease in one patient.

Three of the five patients had MAC antibody titers above the upper limit of measurement (>10 U/mL). At the time of MPO-ANCA positivity, sputum mycobacterial cultures were positive in three patients, including one who was smear-positive. Four patients were MPO-ANCA-positive at the initial ANCA measurement, whereas one patient was initially MPO-ANCA-negative and subsequently developed MPO-ANCA seroconversion during NTM treatment. One patient developed AAV with biopsy-proven pauci-immune crescentic glomerulonephritis and mononeuritis multiplex. During follow-up, MPO-ANCA became negative in two patients, remained positive and gradually increased in two, and decreased but remained positive during AAV treatment in one patient.

### 3.2. Comparison of MPO-ANCA-Positive and MPO-ANCA-Negative Patients

The clinical characteristics of the MPO-ANCA-positive (*n* = 5) and MPO-ANCA-negative (*n* = 44) groups are shown in Table 2. No significant differences were observed between the two groups in terms of age, sex, BMI, smoking history, history of multidrug NTM treatment, systemic immunosuppression at ANCA testing, bilateral lung involvement, NICE score, neutrophil count, serum albumin level, CRP level, or HbA1c level.

In contrast, the MAC antibody titer was significantly higher in the MPO-ANCA-positive group than in the MPO-ANCA-negative group [10.0 (3.89–10.0) vs. 1.92 (0.19–6.19) U/mL, *p* = 0.038]. In a subanalysis restricted to patients with pulmonary MAC disease, the MAC antibody titer also remained significantly higher in the MPO-ANCA-positive group than in the MPO-ANCA-negative group [10.0 (8.08–10.0) vs. 2.32 (0.99–6.94) U/mL, *p* = 0.040].

## 4. Discussion

This study analyzed 49 patients with NTM-PD who underwent testing for MPO-ANCA and PR3-ANCA at our hospital. MPO-ANCA was positive in five patients (10.2%), whereas PR3-ANCA was positive in only one patient (2.0%), indicating that ANCA positivity was mainly observed as MPO-ANCA positivity. This frequency was close to the ANCA-positivity rate of 11.1% reported by Oda et al. in patients with NTM-PD, supporting previous evidence that MPO-ANCA positivity occurs at a certain frequency in patients with NTM-PD [3]. Furthermore, Tamaki et al. reported that among 1204 Japanese individuals undergoing health checkups, the positivity rates for MPO-ANCA and PR3-ANCA were 0.25% and 0.66%, respectively [9]. Although the present study population differed from the health checkup population in terms of background characteristics, such as age, the frequency of MPO-ANCA positivity appeared to be relatively high.

Both host susceptibility and environmental factors may contribute to ANCA positivity. Overall, environmental factors associated with ANCA induction or AAV development include infection, smoking, silica exposure, organic solvents, and drugs [10]. A history of smoking is reportedly associated with AAV, particularly MPO-ANCA-positive AAV, supporting the potential role of inhalational environmental factors in MPO-ANCA-related autoimmunity [11]. 

Respiratory infections have also been associated with the development of MPO-ANCA-positive AAV. In a Swedish population-based case–control study, upper respiratory tract infection and pneumonia were associated with subsequent AAV development, and this association was significant for MPO-ANCA-positive AAV but not for PR3-ANCA-positive AAV [12].

Pulmonary tuberculosis, another pulmonary mycobacterial infection, has also been investigated in relation to ANCA positivity. Previous studies have reported variable ANCA findings in tuberculosis, including MPO-ANCA-dominant [13], PR3-ANCA-dominant [14], and bactericidal/permeability-increasing protein ANCA-dominant [15] patterns. These differences, together with evidence that anti-tuberculosis treatment may affect autoantibody levels, suggest that ANCA profiles in tuberculosis may be influenced by treatment timing, assay method, and patient background [13,14,15,16]. ANCA-positive AAV has also been reported in extrapulmonary tuberculosis, although evidence is limited to case reports [17].

Among chronic pulmonary diseases, reports have accumulated mainly in the field of interstitial pneumonia and pulmonary fibrosis, in which lung disease can precede ANCA positivity or the development of MPA. Kagiyama et al. retrospectively studied 504 patients with idiopathic pulmonary fibrosis and reported that, at diagnosis, 20 patients (4.0%) were MPO-ANCA-positive and 16 patients (3.2%) were PR3-ANCA-positive; during follow-up, among patients who were ANCA-negative at diagnosis, 15 (5.7%) developed MPO-ANCA positivity and 14 (5.3%) developed PR3-ANCA positivity [18]. In that study, rheumatoid factor positivity and elevated erythrocyte sedimentation rate were associated with MPO-ANCA seroconversion [18].

Collectively, these observations suggest that the lung may not merely be a target organ of AAV but may also serve as a local immune environment that contributes to MPO-ANCA positivity [19].

The present study evaluated the NICE score as an indicator of structural lung damage and disease burden. However, no significant difference was observed between the MPO-ANCA-positive and MPO-ANCA-negative groups. This finding suggests that structural lung damage may not directly correspond to MPO-ANCA positivity. However, because the number of MPO-ANCA-positive patients was small, the statistical power may have been insufficient to detect true differences. Although the NICE score [8] evaluates the extent and structural changes in radiographic lung lesions, it does not distinguish between the current viable bacterial burden, active infection, residual structural damage after treatment, and cumulative inflammatory burden over time. Therefore, the NICE score alone may not adequately capture the antigenic stimulation or local pulmonary inflammation that could be involved in MPO-ANCA induction.

In contrast, the MAC antibody titer was associated with MPO-ANCA positivity in this study. MAC antibody is an IgA antibody targeting the glycopeptidolipid (GPL)-core antigen on the surface of MAC organisms and is used as an adjunctive diagnostic marker for pulmonary MAC disease [6]. Anti-GPL-core IgA antibody titers are associated with the extent of radiographic lesions and disease progression requiring treatment [20,21]. Although MAC antibody results may be discordant with culture-based NTM identification in individual cases, the higher titers observed in this study may reflect chronic MAC-related antigenic or inflammatory activity rather than the current viable bacterial burden alone. Thus, although not established as a predictor of MPO-ANCA seroconversion, the MAC antibody titer may serve as an exploratory marker of such activity.

A possible molecular link between chronic pulmonary immune stimulation and MPO-ANCA positivity is that NET-associated MPO may be transferred to myeloid dendritic cells, thereby promoting adaptive autoimmune responses that drive MPO-ANCA production. This, in turn, further amplifies neutrophil activation and NET release [22,23]. Although largely extrapolated from experimental studies of AAV, this proposed feed-forward mechanism may provide a biologically plausible explanation for the induction or persistence of MPO-ANCA in NTM-PD, even when viable NTM are not continuously detected [4,22,23].

Longitudinal findings in this cohort underscore the heterogeneous clinical significance of MPO-ANCA positivity in NTM-PD. Although one patient developed overt AAV, the remaining patients either became MPO-ANCA-negative or had persistent or increasing titers without vasculitic manifestations; in the patient with AAV, titers decreased after treatment. This pattern aligns with reports that ANCA may occur without clinical AAV and that only a subset of MPO-ANCA-positive or seroconverted patients subsequently develop MPA [9,18,19], as well as with the long-term clinical outcomes reported in patients with NTM-PD [3]. Nevertheless, subsequent AAV development in the patients without overt vasculitis cannot be excluded because follow-up duration and ANCA testing intervals were not standardized. Therefore, ANCA trends should be interpreted together with clinical assessment for emerging organ involvement, particularly renal and neurologic manifestations [1,3,19].

This study has several limitations that should be acknowledged. First, this was a single-center retrospective study with a small sample size, particularly for MPO-ANCA-positive patients; therefore, multivariate analysis and establishment of a clear cutoff value for MAC antibody titers were not feasible. Second, MPO-ANCA and PR3-ANCA were not systematically measured in all patients with NTM-PD but were measured based on clinical judgment, mainly in patients with progressive disease. Therefore, the analyzed population may not be representative of the overall NTM-PD population and may have been biased toward patients with more advanced pulmonary disease. Third, the timing and intervals of ANCA testing were not standardized, and preexisting ANCA positivity before the diagnosis or progression of NTM-PD could not be excluded. Furthermore, the potential influence of unmeasured host immune factors could not be assessed. Therefore, this study could not determine the temporal or causal relationship among NTM-PD, MAC antibody titers, MPO-ANCA positivity, and AAV development. Given these limitations, the findings of this study should be interpreted as hypothesis-generating.

## 5. Conclusions

In this exploratory study, MPO-ANCA positivity was observed in a subset of patients with NTM-PD, and higher MAC antibody titers were observed in patients with MPO-ANCA positivity. Although a high MAC antibody titer is not an established marker for predicting MPO-ANCA seroconversion, it may serve as an exploratory indicator of chronic MAC antigen exposure or local pulmonary disease activity. The present findings may increase the clinical awareness of the importance of MPO-ANCA positivity in patients with NTM-PD; however, whether ANCA testing should be performed routinely, which patients should be tested, and how ANCA-positive patients should be followed remain unclear. Prospective studies are needed to clarify the temporal relationships between ANCA seroconversion, changes in MAC antibody titers, pulmonary disease activity, immunological markers, and AAV development in patients with NTM-PD.

## Figures and Tables

**Table 1:** Clinical characteristics and follow-up of patients with MPO-ANCA positivity.

Case	Age, Years	Sex	Smoking History	NTM Species	NTM-PD Duration, Years	MAC Antibody Titer, U/mL	MPO-ANCA Course, U/mL	Follow-Up, Years	Radiographic Pattern	NICE Score	NTM Treatment Status	Sputum Mycobacterial Status	AAV Manifestations, Treatment, and Outcome
1	72	F	No	*M. avium*	40	2.31	4.8 → 2.0	4	Bilateral nodular/bronchiectatic disease	11	CAM monotherapy	Culture-positive	No AAV during follow-up ^‡^
2	80	F	No	*M. avium* *M. intracellulare*	13	>10	783 → ~20 after initiation of AAV treatment	2	Bilateral nodular/bronchiectatic disease	10	RE-CAM for 2.5 years → observation	Negative	Crescentic GN and mononeuritis multiplex; ongoing PSL + RTX; clinically stable
3	81	F	No	*M. kansasii*	8	3.89	7.2 → approximately 10 (gradual increase)	2	Bilateral nodular/bronchiectatic disease	13	HER → EM maintenance	Negative	No AAV during follow-up ^‡^
4	89	F	No	*M. avium*	Unknown	>10	85.3 → approximately 300 (gradual increase)	3	Bilateral fibrocavitary disease	7	Untreated	Culture-positive	No AAV during follow-up ^‡^
5	80	F	Yes	*M. avium*	Unknown	>10	10.1 ^†^ → 0.3	1	Bilateral nodular/bronchiectatic disease	18	RE-CAM + AMK	Smear- and culture-positive	No AAV during follow-up ^‡^

AAV, anti-neutrophil cytoplasmic antibody-associated vasculitis; AMK, amikacin; CAM, clarithromycin; EM, erythromycin; F, female; GN, glomerulonephritis; HER, isoniazid, ethambutol, and rifampicin; MAC, *Mycobacterium avium* complex; MPO-ANCA, myeloperoxidase anti-neutrophil cytoplasmic antibody; NICE, nodule, infiltration/consolidation, cavity, and ectasis; NTM, nontuberculous mycobacteria; NTM-PD, nontuberculous mycobacterial pulmonary disease; PSL, prednisolone; RE-CAM, rifampicin, ethambutol, and clarithromycin; RTX, rituximab. The detailed clinical course of Case 2 has been reported previously [2]. None of the five patients had a diagnosed autoimmune disease before MPO-ANCA positivity. Case 3 had an antinuclear antibody titer of 1:640 and was positive for anti-SSA/Ro and anti-SSB/La antibodies, without sicca symptoms or a diagnosis of Sjögren’s syndrome. ^†^ Case 5 was MPO-ANCA-negative at the initial measurement, seroconverted to 10.1 U/mL during NTM treatment, and subsequently reverted to negative status. ^‡^ In Cases 1, 3, 4, and 5, no clinical evidence suggestive of overt AAV, including renal involvement, peripheral neuropathy, pulmonary hemorrhage, or other systemic vasculitic manifestations, was observed throughout the follow-up period after the first detection of MPO-ANCA positivity.

**Table 2:** Clinical Factors Associated with MPO-ANCA Positivity in Patients with NTM-PD.

Variable	MPO-ANCA-Positive (*n* = 5)	MPO-ANCA-Negative (*n* = 44)	*p* Value
Age, years	80 (80–81)	74 (66–80.5)	0.146
Female sex, *n*/*N* (%)	5/5 (100)	32/43 (74.4)	0.576
BMI, kg/m^2^	20.75 (20.05–21.38)	18.90 (16.60–20.80)	0.225
Smoking history, *n*/*N* (%)	1/5 (20.0)	10/36 (27.8)	1.000
History of multidrug NTM treatment, *n*/*N* (%)	3/5 (60.0)	10/40 (25.0)	0.136
Systemic immunosuppression at ANCA testing, *n*/*N* (%)	0/5 (0)	1/44 (2.3)	1.000
Bilateral lung involvement, *n*/*N* (%)	5/5 (100)	33/43 (76.7)	0.569
NICE score	13 (11–15)	7 (3–15)	0.141
Neutrophil count, cells/µL	3730 (3630–3980)	3960 (3035–4680)	0.673
Serum albumin, g/dL	3.8 (2.7–4.1)	3.9 (3.8–4.1)	0.591
CRP, mg/dL	0.95 (0.11–1.88)	0.15 (0.04–0.57)	0.182
HbA1c, %	5.7 (5.7–5.8)	5.9 (5.7–5.93)	0.431
MAC antibody titer, U/mL	10.0 (3.89–10.0)	1.92 (0.19–6.19)	0.038

Data are presented as medians with interquartile ranges or *n*/*N* (%). ANCA, anti-neutrophil cytoplasmic antibody; BMI, body mass index; CRP, C-reactive protein; MAC, *Mycobacterium avium* complex; MPO-ANCA, myeloperoxidase anti-neutrophil cytoplasmic antibody; NICE, nodule, infiltration/consolidation, cavity, and ectasis; NTM, nontuberculous mycobacteria; NTM-PD, nontuberculous mycobacterial pulmonary disease. *p*-values were calculated using the Mann–Whitney U test for continuous variables and Fisher’s exact test for categorical variables. The denominators varied among the variables because of missing data.

## Data Availability

The data presented in this study are available upon reasonable request from the corresponding author. The data are not publicly available due to privacy and ethical restrictions.

## Abbreviations

AAV	ANCA-associated vasculitis
ANCA	anti-neutrophil cytoplasmic antibody
BMI	body mass index
CLEIA	chemiluminescent enzyme immunoassay
CRP	C-reactive protein
GPL	glycopeptidolipid
IgA	immunoglobulin A
MAC	*Mycobacterium avium* complex
MPA	microscopic polyangiitis
MPO-ANCA	myeloperoxidase anti-neutrophil cytoplasmic antibody
NET	neutrophil extracellular trap
NICE	nodule, infiltration/consolidation, cavity, and ectasis
NTM	nontuberculous mycobacteria
NTM-PD	nontuberculous mycobacterial pulmonary disease
PR3-ANCA	proteinase 3 anti-neutrophil cytoplasmic antibody
